# Three-segment lateral δ-doping redistribution in GaAs pHEMTs for gate–drain field relief and improved off-state robustness

**DOI:** 10.1038/s41598-026-50321-8

**Published:** 2026-04-29

**Authors:** Shishi Liao, Jin Xu, Jian Yang, Qiuhong Xie, Qi Jiang

**Affiliations:** 1 School of Electrical and Electronic Information Engineering, Sichuan University Jinjiang College, Meishan, 620860 China; 2 School of Computer Science, Sichuan University Jinjiang College, Meishan, 620860 China; 3 Intelligent Digital Media Technology Key Laboratory of Sichuan Province, Chengdu, 610054 China

**Keywords:** GaAs pHEMTs, δ-doping, Lateral redistribution, Electric-field crowding, Robustness–performance trade-off, Energy science and technology, Engineering, Physics

## Abstract

In GaAs pseudomorphic high-electron-mobility transistors (pHEMTs) for high-power and high-linearity radio-frequency (RF) applications, significant lateral electric-field crowding at the gate–drain edge is a common issue under high drain bias, particularly during off-state and semi-off-state operation. Alleviating this peak field, however, typically leads to an increase in access resistance and degrades key RF figures of merit, resulting in an inherent robustness–performance trade-off. This work proposes a three-segment lateral δ-doping redistribution strategy in which the gate-under segment is kept unchanged, the drain-side segment is progressively reduced, and the redistributed dose is compensated by increasing the source-side segment, thereby approximately conserving the total length-weighted lateral δ-dose. Two-dimensional TCAD simulations were performed for four schemes (K1P0, K0P8, K0P6, and K0P4) using identical device geometry, material composition, and physical-model settings. DC and RF small-signal metrics were evaluated alongside an off-state robustness assessment. Electric-field mapping under a common reference bias indicates a systematic reduction in the peak lateral electric field near the gate–drain edge as the drain-side δ-doping is weakened. To enable a reproducible robustness comparison in a simulation-based study, a practical robustness metric, *V*_crit_, is defined as the applied drain voltage at which |*I*_D_| reaches 1 × 10^− 5^ A under off-state stress (*V*_G_= -3 V). *V*_crit_ increases monotonically from 11.99 V (K1P0) to 13.28 V (K0P8), 15.41 V (K0P6), and 19.06 V (K0P4), representing improvements of 10.8%, 28.5%, and 58.9%, respectively. This gain in robustness comes at the expense of DC/RF performance: the width-normalized on-resistance (*R*_on·W_) increases by up to 45.4%, and the transition frequency measured at the 0.3*I*_DSS_ operating point decreases by up to 11.7%. These simulation-based results quantify the robustness–performance trade-off and provide a comparative basis for evaluating lateral δ-doping redistribution within the explored design space.

## Introduction

GaAs pseudomorphic high-electron-mobility transistors (pHEMTs) are widely employed in radio-frequency (RF) and microwave front-end circuits owing to their high electron mobility and excellent small-signal performance^1–5^. For high-power and high-linearity applications, robustness under elevated drain bias—particularly under off-state and semi-off-state conditions—is critically important. A major limitation arises from pronounced electric-field crowding near the gate–drain edge, which can induce gate and drain leakage, activate trap-related degradation mechanisms, and accelerate reliability wear-out, thereby constraining long-term stability and high-voltage operating margins^6–8^.

Conventional field-relief approaches include increasing the gate–drain spacing, adopting field-plate structures, optimizing passivation and surface states, and tailoring barrier/channel designs. While effective, these methods often introduce additional process complexity and/or incur penalties in on-resistance and RF figures of merit. It is therefore of practical interest to identify process-compatible design strategies that can redistribute the channel potential and alleviate the gate–drain peak electric field while allowing the associated performance trade-off to be quantitatively evaluated^9–11^.

δ-doping engineering offers a direct means to control access-region electrostatics. In particular, a laterally segmented δ-doping redistribution strategy can modulate the access-region charge profile, the longitudinal potential drop, and ultimately the gate–drain edge field crowding. However, systematic quantitative studies of controlled lateral δ-doping redistribution in GaAs pHEMTs remain limited, especially for the case in which the gate-under segment is fixed to preserve the intrinsic channel electrostatics, while the remaining lateral δ-doping is redistributed under an approximately conserved total length-weighted lateral δ-dose.

Compared with prior studies that mainly focus on structural field-management approaches or on general δ-doping/profile variation for RF or linearity optimization^9–11^, the present work addresses a more specific question: how controlled lateral δ-doping redistribution affects field relief and the associated electrical trade-off when the gate-under segment is kept fixed. The contribution of this study therefore lies in a controlled comparative framework, in which the gate-under segment is unchanged, the source-side and drain-side δ-doping are systematically redistributed under an approximately conserved total length-weighted lateral δ-dose, and the resulting impact on electric-field redistribution, DC/RF penalties, and the comparative off-state robustness metric *V*_crit_ is evaluated under otherwise identical geometry, epitaxial structure, and model settings.

In this work, we propose a three-segment lateral δ-doping redistribution strategy. The δ-doping concentration beneath the gate-foot region is held constant, while the drain-side segment is progressively reduced and the corresponding dose is transferred to the source-side segment, thereby approximately conserving the total length-weighted lateral δ-dose. This study focuses on a controlled lateral redistribution framework in which the gate-under segment is fixed and the redistribution effect is isolated under otherwise identical geometry, material composition, and model settings. We quantify the resulting changes in DC and RF small-signal metrics, correlate them with mitigation of the electric field near the gate–drain edge, and establish a quantitative robustness–performance trade-off relationship. To enable a reproducible robustness comparison in a simulation-based study, we introduce a practical off-state robustness metric, *V*_crit_, defined as the applied drain voltage at which $\:\mid\:{I}_{\mathrm{D}}\mid\:$ reaches 1 × 10^− 5^ A under a fixed gate-off bias (*V*_G_ = -3 V)^12–14^.

The remainder of this paper is organized as follows. The section “Device structure and three-segment lateral δ-doping redistribution” describes the device structure and the proposed three-segment δ-doping schemes. The section “TCAD methodology and metric definitions” summarizes the simulation methodology, bias conditions, and metric definitions. The section “Results and discussion” presents DC and RF results, electric-field analysis, and robustness trends, followed by a discussion of the associated trade-offs. The section “Conclusions” concludes the paper.

## Device structure and three-segment lateral δ-doping redistribution

**Fig. 1:**
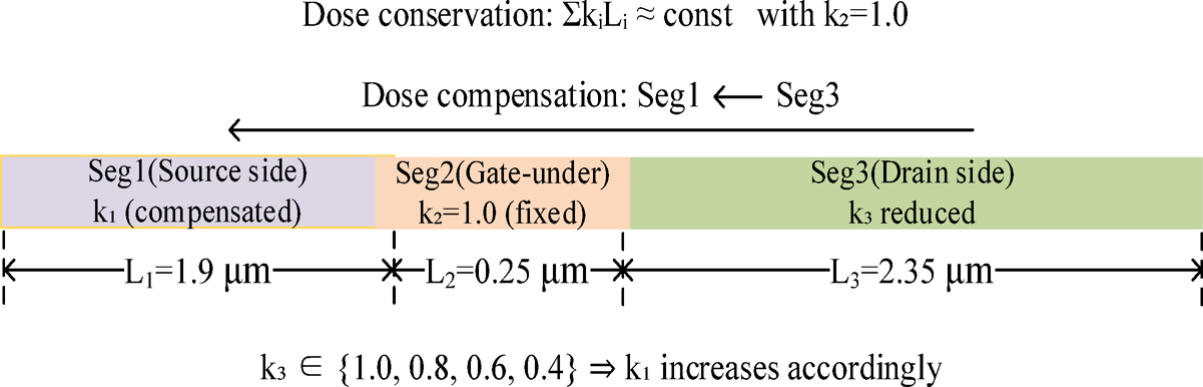
Schematic of the three-segment lateral δ-doping redistribution concept, illustrating the approximate dose-conservation rule Σk_i_L_i_ ≈ const with k₂ = 1.0. The dose factor in the drain-side segment (k₃) is reduced stepwise, and the compensated dose is transferred to the source-side segment (k₁ increases accordingly).

**Fig. 2:**
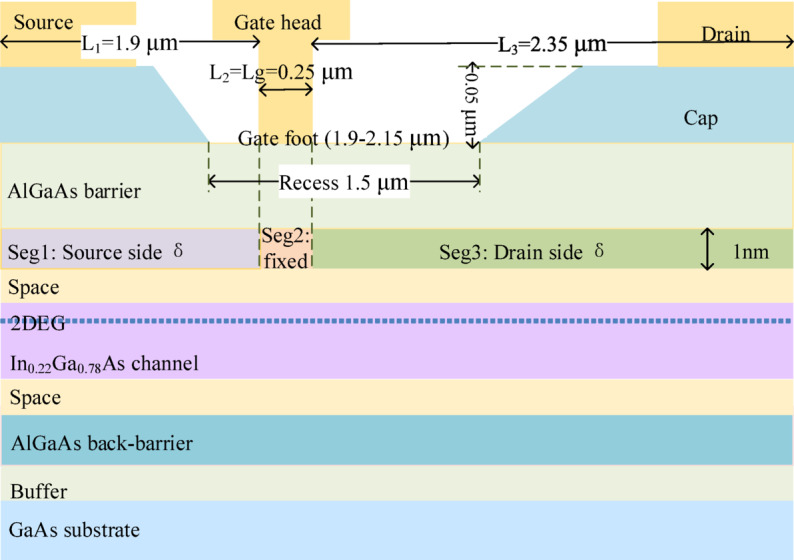
Two-dimensional cross-section of the modeled GaAs pHEMT, showing the epitaxial layer stack and key geometric dimensions.

**Fig. 3:**
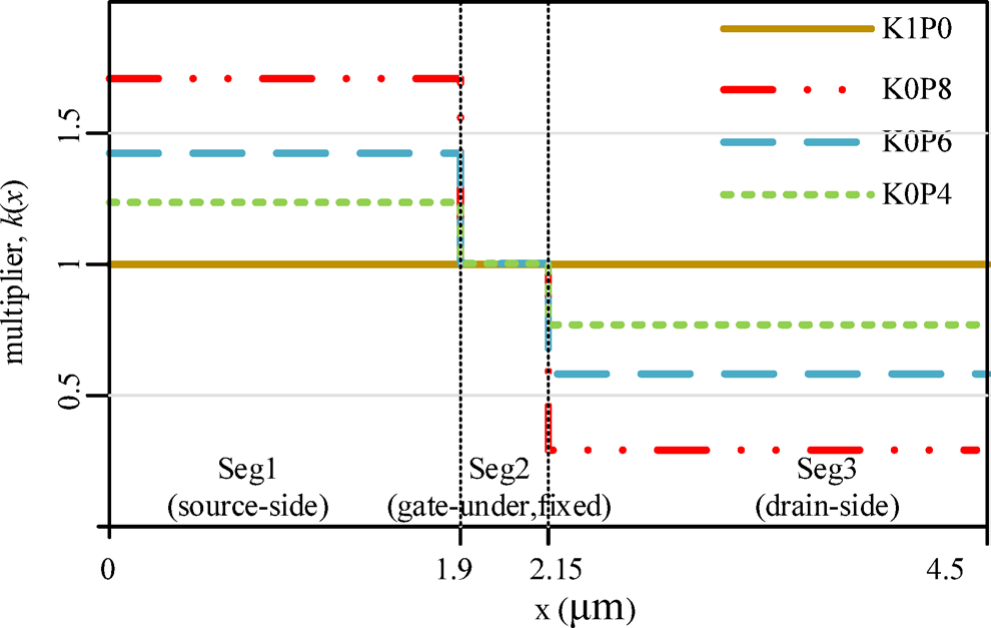
Lateral three-segment δ-doping definition and comparison of the segment-wise δ-doping multipliers *k*(*x*) for the four schemes (K1P0, K0P8, K0P6, K0P4). The Seg2 (gate-under) multiplier is fixed at $\:{k}_{2}=1.0$.

This section defines the device under study and the proposed three-segment lateral δ-doping redistribution strategy. To enable a controlled and interpretable comparison, all cases employ an identical device geometry, epitaxial layer stack, and non-δ parameters; only the segment-wise δ-doping levels are intentionally varied. As illustrated in Fig. 1, the δ-doping distribution along the access region is divided into three lateral segments: a source-side segment (Seg1, length L₁ = 1.9 μm, dose factor k₁), a gate-under segment (Seg2, L₂ = 0.25 μm, k₂ fixed at 1.0), and a drain-side segment (Seg3, L₃ = 2.35 μm, dose factor k₃). The redistribution follows a dose-conservation principle: the total length-weighted lateral δ-dose is approximately conserved (Σk_i_L_i_ ≈ const) by reducing k₃ in predefined steps (1.0, 0.8, 0.6, 0.4) and transferring the removed dose to Seg1 (i.e., increasing k₁ accordingly). Figure 2 shows the two-dimensional device cross-section and layer stack, and Fig. 3, together with Table 2, summarizes the resulting segment-wise δ-doping profiles for all schemes. The fixed structural parameters are listed in Table 1^15,16^.

### Device structure and δ-like doping implementation

Figure 2 shows the modeled GaAs pHEMT cross-section. A recessed T-gate is adopted to enhance electrostatic control. The epitaxial stack follows a conventional GaAs/AlGaAs/InGaAs pHEMT material system, including an Al_0.22_Ga_0.78_As barrier and an In_0.22_Ga_0.78_As channel, with spacers, back-barrier/buffer, and substrate layers underneath. Source and drain ohmic contacts are placed on the cap layer, and the gate foot defines the intrinsic gate-controlled region.

To represent δ-doping in a numerically stable and TCAD-friendly manner^17^, a finite-thickness “δ slab” is used to approximate sheet-like doping. Specifically, a 1-nm-thick donor-doped layer is introduced at a fixed vertical location, and its volume concentration is used as the tuning parameter. The δ-slab thickness and vertical placement are kept identical across all cases, ensuring that any performance/robustness change can be attributed to the lateral redistribution (x-direction segmentation) rather than to variations in vertical placement or thickness^18–20^. The fixed geometry, layer thicknesses, and key x/y definitions are summarized in Table 1.

**Table 1:** Fixed device geometry and structural parameters (all schemes share the same parameters; only the segment-wise δ-doping levels vary).

Category	Parameter	Value
Material system	Barrier / Channel	Al_0.22_Ga_0.78_As / In_0.22_Ga_0.78_As
Gate	T-gate head (x-range)	x = 1.55–2.45 μm
Gate	Gate foot length $\:{L}_{g}$	0.25 μm (x = 1.90–2.15 μm)
Geometry	Recess depth	0.05 μm
Contacts	Source metal (x-range)	x = 0–1.0 μm
Contacts	Drain metal (x-range)	x = 3.5–4.5 μm
Layer thickness	AlGaAs barrier	0.03 μm (y = 0.05–0.08 μm)
Layer thickness	Spacer layers	0.003 μm each (3 nm)
Layer thickness	InGaAs channel	0.014 μm (14 nm)
δ implementation	δ-slab thickness $\:{t}_{\delta\:}$	0.001 μm (1 nm)
δ implementation	δ-slab vertical location	y = 0.080–0.081 μm
Segmentation	δ segments (x-ranges)	Seg1: 0–1.90 μm; Seg2: 1.90–2.15 μm; Seg3: 2.15–4.50 μm

### Definition of Seg1/Seg2/Seg3 partitions in the δ-doping slab

The δ-doping slab is laterally partitioned into three segments along the channel direction (Fig. 1) to enable access-region charge engineering while preserving the intrinsic gate-controlled electrostatics. The three segments are defined as follows:

- Seg1(source-side δ segment): $\:x\in\:[0,\:1.9]\text{}{\upmu\:}\mathrm{m}$
- Seg2 (gate-under δ segment): $\:x\in\:\left[1.9,\:2.15\right]\:{\upmu\:}\mathrm{m}$ (aligned with the gate foot).
- Seg3 (drain-side δ segment): $\:x\in\:\left[\mathrm{2.15,4.5}\right]\:{\upmu\:}\mathrm{m}$

All three segments share the same δ-slab vertical location $\:y\in\:[0.080,\:0.081]\text{}{\upmu\:}\mathrm{m}$ and thickness $\:{t}_{\delta\:}=1\:$ nm. A key design principle of this study is to keep Seg2 fixed for all schemes, so that the intrinsic gate-under electrostatics (and thus the channel opening condition) remains as comparable as possible among cases. Under this constraint, Seg1 and Seg3 act as the primary knobs to redistribute access-region charge and reshape the longitudinal potential drop, which is expected to directly influence electric-field crowding near the gate–drain edge under high drain bias and off-/semi-off conditions. The resulting piecewise-constant lateral profiles are illustrated in Fig. 3.

### Redistribution schemes (K1P0/K0P8/K0P6/K0P4) and dose-conservation constraint

Four redistribution schemes are constructed by adjusting the peak donor concentration in Seg1/Seg2/Seg3 while keeping all non-δ parameters unchanged. Let $\:{N}_{\delta\:,0}$ denote the baseline peak donor concentration in the δ slab, and define dimensionless multipliers ($\:{k}_{1},\:{k}_{2},\:{k}_{3})\:$ such that the segment-wise peak concentrations are:

1 $$\:{N}_{\delta\:,i}={k}_{i}{\hspace{0.17em}}{N}_{\delta\:,0},i\in\:\{\mathrm{1,2},3\},$$

In this work, the gate-under segment multiplier is fixed at $\:{k}_{2}=1.0$, while the drain-side multiplier *k*₃ is progressively reduced to implement drain-side δ-doping weakening. To isolate the effect of lateral redistribution from a change in the total incorporated dose, an approximately length-weighted dose-conservation constraint is imposed:

2 $$\:{k}_{1}{L}_{1}+{k}_{2}{L}_{2}+{k}_{3}{L}_{3}\approx\:{L}_{1}+{L}_{2}+{L}_{3},$$

where $\:\:\left({L}_{1},\:\:{L}_{2},\:\:{L}_{3}\right)=\left(1.9,\:\:0.25,\:\:2.35\right)$ µm are the segment lengths. With $\:{k}_{2}=1.0$ and a prescribed $\:{k}_{3}$, the corresponding source-side multiplier $\:{k}_{1}$ is obtained as:

3 $$\:{k}_{1}=\frac{({L}_{1}+{L}_{2}+{L}_{3})-{k}_{2}{L}_{2}-{k}_{3}{L}_{3}}{{L}_{1}},$$

Accordingly, the K1P0 scheme serves as the segmented-uniform baseline with $\:\left({k}_{1},{k}_{2},{k}_{3})=(\mathrm{1.0,1.0,1.0}\right)$. The K0P8, K0P6, and K0P4 schemes progressively reduce *k*_3_ to 0.8, 0.6, and 0.4, respectively, and increase *k*₁ accordingly to maintain the approximate dose conservation defined by Eq. (2). The segment-wise multipliers for all schemes are summarized in Table 2, and the corresponding lateral doping profiles are shown in Fig. 3. This approach establishes a controlled design variable—the Seg1-to-Seg3 dose ratio under a fixed Seg2—to systematically investigate the trade-offs between robustness and performance induced by lateral δ-doping redistribution.

**Table 2:** Three-segment lateral δ-doping redistribution schemes defined by segment-wise peak-concentration multipliers ($\:{k}_{1},{k}_{2},{k}_{3}$) relative to the baseline peak concentration $\:{N}_{\delta\:,0}$ within the 1-nm δ slab. Seg2 is fixed at $\:{k}_{2}=1.0$.

Case	Seg1 $\:{\boldsymbol{k}}_{1}$ (0–1.90 μm)	Seg2 $\:{\boldsymbol{k}}_{2}$ (1.90–2.15 μm, fixed)	Seg3 $\:{\boldsymbol{k}}_{3}$ (2.15–4.5 μm)
K1P0	1	1	1
K0P8	1.247	1	0.8
K0P6	1.495	1	0.6
K0P4	1.742	1	0.4

The δ slab is located at y = 0.080–0.081 μm with thickness $\:{t}_{\delta\:}=1$ nm. Only the multipliers ($\:{k}_{1},{k}_{2},{k}_{3}$) are varied among schemes; all other geometry and layer parameters are fixed as in Table 1.

## TCAD methodology and metric definitions

### Meshing strategy and numerical settings

All simulations are performed on a two-dimensional cross-section of the GaAs pHEMT. Special care is required in mesh generation due to (i) the representation of the δ-doping by an ultra-thin (1 - nm) slab and (ii) strong electric-field and carrier-concentration gradients near the recessed gate edges, particularly at the gate–drain side under high drain bias. Therefore, mesh refinement is applied in the following critical regions:

- δ-slab and adjacent heterointerfaces: The 1-nm δ-slab and the adjacent spacer/channel interfaces are refined to resolve the abrupt vertical doping discontinuity and the associated charge confinement.
- Recessed gate region: Fine meshing is employed around the recess corners and beneath the gate foot to accurately capture steep electrostatic potential gradients.
- Gate–drain edge vicinity: The drain-side gate edge is refined to resolve the electric-field crowding region that governs off-state and semi-off-state high-voltage behavior.

In addition, the lateral segmentation boundaries (at x = 1.90 μm and x = 2.15 μm) are meshed with local refinement to avoid numerical artifacts due to abrupt step changes in the segment-wise δ-doping concentration.

**Fig. 4:**
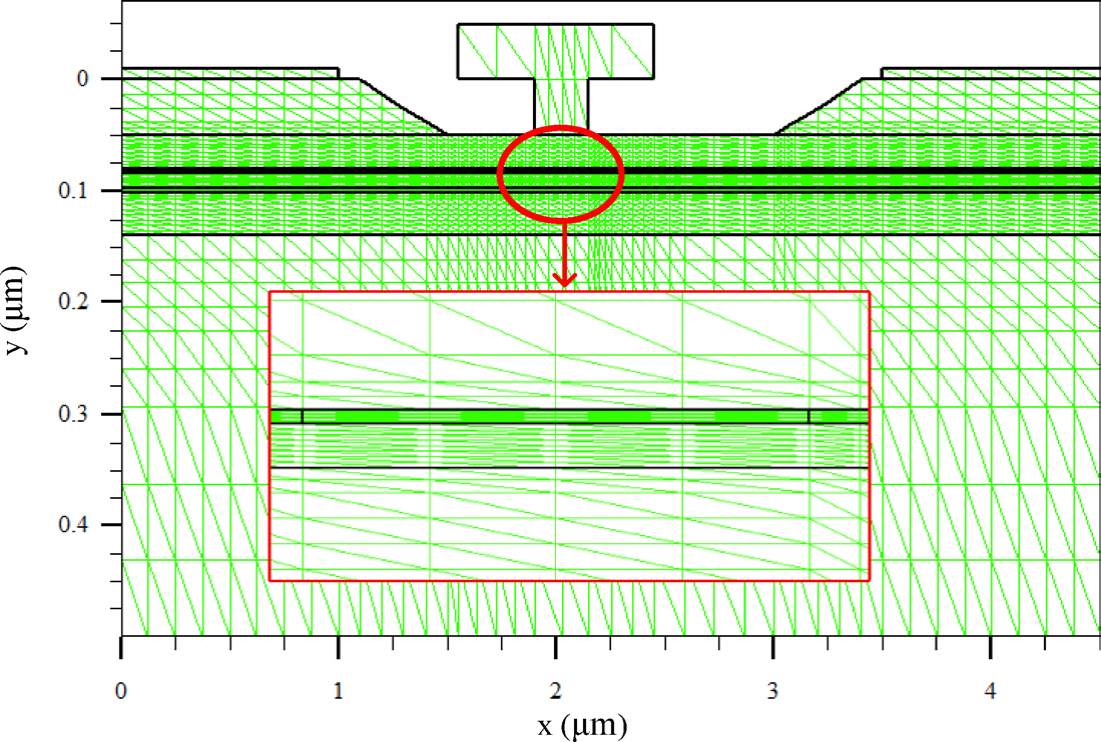
Mesh layout used in the TCAD simulation, highlighting the locally refined regions around the δ-slab, the recessed gate, the gate–drain edge, and the lateral segmentation boundaries.

To maintain comparative consistency, identical meshing rules and numerical-solver settings are used for all redistribution schemes. Figure 4 shows the mesh layout and the locally refined regions around the δ-slab, the recessed gate, the gate–drain edge, and the lateral segmentation boundaries^21–23^.

### Physical models and boundary conditions

The device is simulated within the drift–diffusion framework, which provides a computationally efficient and widely adopted baseline for comparative TCAD studies of design variations in GaAs pHEMTs. Ohmic boundary conditions are applied at the source and drain contacts, while the recessed gate is modeled as a Schottky contact, thereby enabling a physical description of gate electrostatics and gate leakage under reverse bias.

To model carrier multiplication in the localized high‑field regions pertinent to off‑state stress, an impact‑ionization (avalanche) model is activated. It is well‑recognized that the absolute avalanche breakdown voltages predicted by TCAD can be sensitive to the choice and parameterization of the impact‑ionization model, as well as to leakage paths influenced by traps and numerical‑convergence strategies. Therefore, this work does not aim to provide a calibrated absolute breakdown voltage. Instead, to enable a reproducible and scheme‑comparative assessment, a practical off‑state robustness metric is defined based on a fixed drain‑current criterion under a fixed gate‑off bias (The section  “Off-state Robustness Definition and Extraction of *V*_crit_”).

Trap-related effects are therefore not treated here as an independent study variable; instead, the present analysis focuses on the comparative impact of lateral δ-doping redistribution under otherwise identical simulation conditions. Physically, traps may modify charge capture/release in the high-field gate–drain region, thereby affecting leakage onset, current-collapse-related behavior, and the apparent robustness margin. Since trap parameters were not independently varied in the present study, these effects are not resolved here, and the results should be interpreted as comparative rather than calibrated absolute predictions. This approach provides stable comparisons across redistribution schemes while remaining aligned with the core engineering objective of suppressing gate–drain field crowding and delaying leakage-current escalation.

### Bias conditions and extraction of DC and small-signal metrics

#### Output characteristics and on‑resistance extraction

DC output characteristics (*I*_D_-*V*_D_) are simulated by sweeping the drain bias under several fixed gate voltages. To extract the on-resistance (*R*_on_) from the linear region with improved robustness against numerical noise, an extended *I*_D_-*V*_D_ sweep at *V*_G_ = 0 V is performed. *R*_on_ is then computed using a two-point slope between *V*_D_= 0.1 V and 0.2 V:

4 $$\:{R}_{\mathrm{o}\mathrm{n}}\approx\:\frac{{\Delta\:}{V}_{\mathrm{D}}}{{\Delta\:}{I}_{\mathrm{D}}}=\frac{0.2-0.1}{{I}_{\mathrm{D}}\left(0.2\right)-{I}_{\mathrm{D}}\left(0.1\right)},$$

To enable a width‑independent comparison, the width‑normalized on‑resistance is reported, following the extraction procedure in the simulation workflow:

5 $$\:{R}_{\mathrm{o}\mathrm{n}\cdot\mathrm{W}}={R}_{\mathrm{o}\mathrm{n}}\times\:{10}^{3}({\Omega\:}\cdot\:\mathrm{m}\mathrm{m}),\:$$

#### Transfer characteristics and small‑signal parameter extraction

Transfer characteristics (*I*_D_-*V*_G_) are simulated at a fixed drain bias of *V*_D_= 2.0 V. The gate voltage is swept from 0 V to − 1.5 V with a constant step size (0.01 V in magnitude). Small-signal quantities are extracted concurrently by enabling AC analysis at a low frequency (1 MHz) during the *I*_D_-*V*_G_ sweep, which yields the gate-source capacitance *C*_gs_ (*V*_G_), gate-drain capacitance *C*_gd_ (*V*_G_), and transconductance *g*_m_ (*V*_G_).

The transconductance is defined as:

6 $$\:{g}_{\mathrm{m}}\left({V}_{\mathrm{G}}\right)=\frac{\partial\:{I}_{\mathrm{D}}}{\partial\:{V}_{\mathrm{G}}},$$

Both its peak value (*g*_m, max_) and its value at *V*_G_ = 0 V are reported as needed.

The saturation current is defined consistently as:

7 $$\:{I}_{\mathrm{D}\mathrm{S}\mathrm{S}}={I}_{\mathrm{D}}({V}_{\mathrm{G}}=0,{\mathrm{V}}_{\mathrm{D}}=\mathrm{2.0}{\:V)}\mathrm{,}$$

A representative RF-relevant operating point is defined at:

8 $$I_{{{\mathrm{D}},{\mathrm{OP}}}} = 0.3\,I_{{{\mathrm{DSS}}}} ,$$

The corresponding gate bias *V*_G, OP_ at this operating point is identified from the transfer curve.

#### RF-relevant metrics: *f*_T_, estimated *f*_max_, and MAG/MSG

The current-gain cutoff frequency (*f*_T_) is calculated using the standard small-signal relation^24^:

9 $$f_{{\mathrm{T}}} = \frac{{g_{{\mathrm{m}}} }}{{2\pi \,\left( {C_{{{\mathrm{gs}}}} + C_{{{\mathrm{gd}}}} } \right)}}$$

and is reported specifically at the operating point defined by *I*_D, OP_= 0.3*I*_DSS_. In this work, the reported metric is denoted as *f*_T_@0.3*I*_DSS_, which is consistent with the extraction procedure in the simulation workflow.

In addition to *f*_T_@0.3*I*_DSS,_ two supplementary RF-related metrics are also included for comparative purposes. The estimated *f*_max_@0.3*I*_DSS_ is extracted from the 0 dB crossing of the unilateral power gain under the same operating point. Owing to the simplified small-signal extraction framework adopted here, this quantity is used as a comparative indicator rather than a calibrated absolute maximum oscillation frequency. In addition, the maximum available gain / maximum stable gain (MAG/MSG) at 10 GHz is evaluated under the same operating point to provide a gain-related comparison among the redistribution schemes.

#### Electric‑field mapping

To directly compare gate–drain edge field crowding under identical external conditions, two-dimensional electric-field distributions are evaluated under a common reference bias^25^. The key quantity analyzed in this work is the lateral electric-field component *E*_x_ along the channel direction. The peak value near the gate–drain edge is extracted from the field map as:

10 $$E_{{{\mathrm{x}},{\mathrm{max}}}} = {\mathrm{max}}\left\{ {E_{{\mathrm{x}}} {\text{ in the gate}} - {\mathrm{drain}}~{\mathrm{edge}}~{\mathrm{vicinity}}} \right\}$$

The same field-probing region and bias condition are applied to all schemes to ensure comparability.

### Off-state robustness definition and extraction of *V*_crit_

Directly reporting an absolute avalanche breakdown voltage from TCAD simulations can be ambiguous, as the predicted breakdown point may depend significantly on the choice and parameterization of the impact-ionization model, underlying leakage mechanisms, and numerical-convergence behavior. To provide a reproducible and engineering-relevant robustness comparison suitable for a simulation-based study, this work defines an off-state drain robustness voltage (*V*_crit_) using a fixed drain-current criterion under a fixed gate-off bias^26–30^:

11 $$\:{V}_{\mathrm{c}\mathrm{r}\mathrm{i}\mathrm{t}}\equiv\:{V}_{\mathrm{D}}\text{}\mathrm{such}\text{}\mathrm{t}\mathrm{hat}\text{}\mid\:{I}_{\mathrm{D}}\mid\:=1\times\:{10}^{-5}\:\mathrm{A},\:\:\:\mathrm{u}\mathrm{n}\mathrm{d}\mathrm{e}\mathrm{r}\:{V}_{G}=-3\:\mathrm{V}$$

**Fig. 5:**
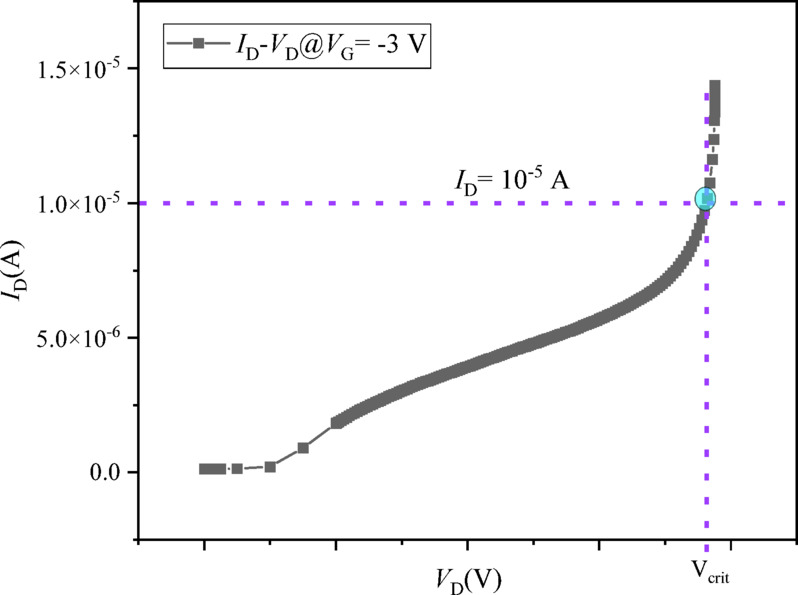
Illustrative off-state *I*_*D*_*–V*_D_ characteristics for the four redistribution schemes, showing the extraction of *V*_crit_ at the |*I*_D_| = 10^−5^ A threshold (dashed horizontal line).

Here, *V*_D_ denotes the externally applied drain bias. In practice, *V*_crit_ is obtained directly from the simulated off-state *I*_D_–*V*_D_ characteristic by applying the same current threshold to all schemes (Fig. 5). This metric quantifies the applied drain-bias level required to reach a specified leakage-current level under a fixed gate-off condition, thereby serving as a consistent comparative measure of the “high-voltage robustness margin” across different lateral δ-doping redistribution schemes.

The key metrics for DC, small-signal/RF, electric-field, and robustness characteristics discussed above, along with extraction rules, and units, are summarized in Table 3.

**Table 3:** Definitions and extraction rules for DC, small-signal/RF, electric‑field, and robustness metrics.

Category	Metric	Symbol	Extraction/definition	Unit
DC	Saturation current	*I*_DSS_	$\:{I}_{\mathrm{D}}\:$ at *V*_G_ = 0 V from the *I*_D_–*V*_G_ sweep (*V*_D_= 2 V)	mA/mm
DC	Threshold voltage	*V*_T_	Extracted from *I*_D_–*V*_G_ at *V*_D_= 2 V using one fixed criterion for all schemes	V
DC	Width-normalized on-resistance (at *V*_G_=0 V)	*R*_on·W_	Two-point slope from *I*_D_–*V*_D_ at *V*_G_ =0 V, normalized to 1-mm gate width	Ω·mm
DC incremental	Transconductance	$\:{g}_{\mathrm{m}}$	$\:{g}_{\mathrm{m}}=\partial\:{I}_{\mathrm{D}}/\partial\:{V}_{\mathrm{G}}$ from *I*_D_–*V*_G_ at *V*_D_= 2 V; report g_m, max_ as needed	mS/mm
AC small-signal	Capacitances	$\:{C}_{\mathrm{g}\mathrm{s}},\:{C}_{\mathrm{g}\mathrm{d}}$	Extracted via AC analysis at 1 MHz during the *I*_D_–*V*_G_ sweep	F/µm
RF-relevant	Cutoff frequency at 0.3*I*_DSS_	$\:{f}_{\mathrm{T}}@0.3{I}_{\mathrm{D}\mathrm{S}\mathrm{S}}$	Calculated from$\:{f}_{T}={g}_{m}/\left[2\pi\:\right({C}_{gs}+{C}_{gd}\left)\right]$ evaluated at the operating point defined by $\:{I}_{D}=0.3{I}_{DSS}$	GHz
RF-relevant	Estimated maximum oscillation frequency at 0.3*I*_DSS_	Estimated $\:{f}_{max}@0.3{I}_{\mathrm{D}\mathrm{S}\mathrm{S}}$	Extracted from the 0 dB crossing of the unilateral power gain under the same operating point defined by $\:{I}_{D}=0.3{I}_{DSS}$	GHz
RF-relevant	Gain-related metric at 10 GHz	MAG/MSG @ 10 GHz	Evaluated at 10 GHz under the same operating point defined by $\:{I}_{D}=0.3{I}_{DSS}$	dB
Field	Peak lateral electric field	*E*_x, max_	Maximum *E*_x_ near the gate–drain edge under a common reference bias	V/cm
Robustness	Off-state drain robustness voltage	$\:{V}_{\mathrm{c}\mathrm{r}\mathrm{i}\mathrm{t}}$	Applied *V*_D_ at which |*I*_D_| = 1 × 10^−^⁵ A under *V*_G_ = -3 V	V

## Results and discussion

### DC characteristics: output, transfer, and transconductance behavior

**Fig. 6:**
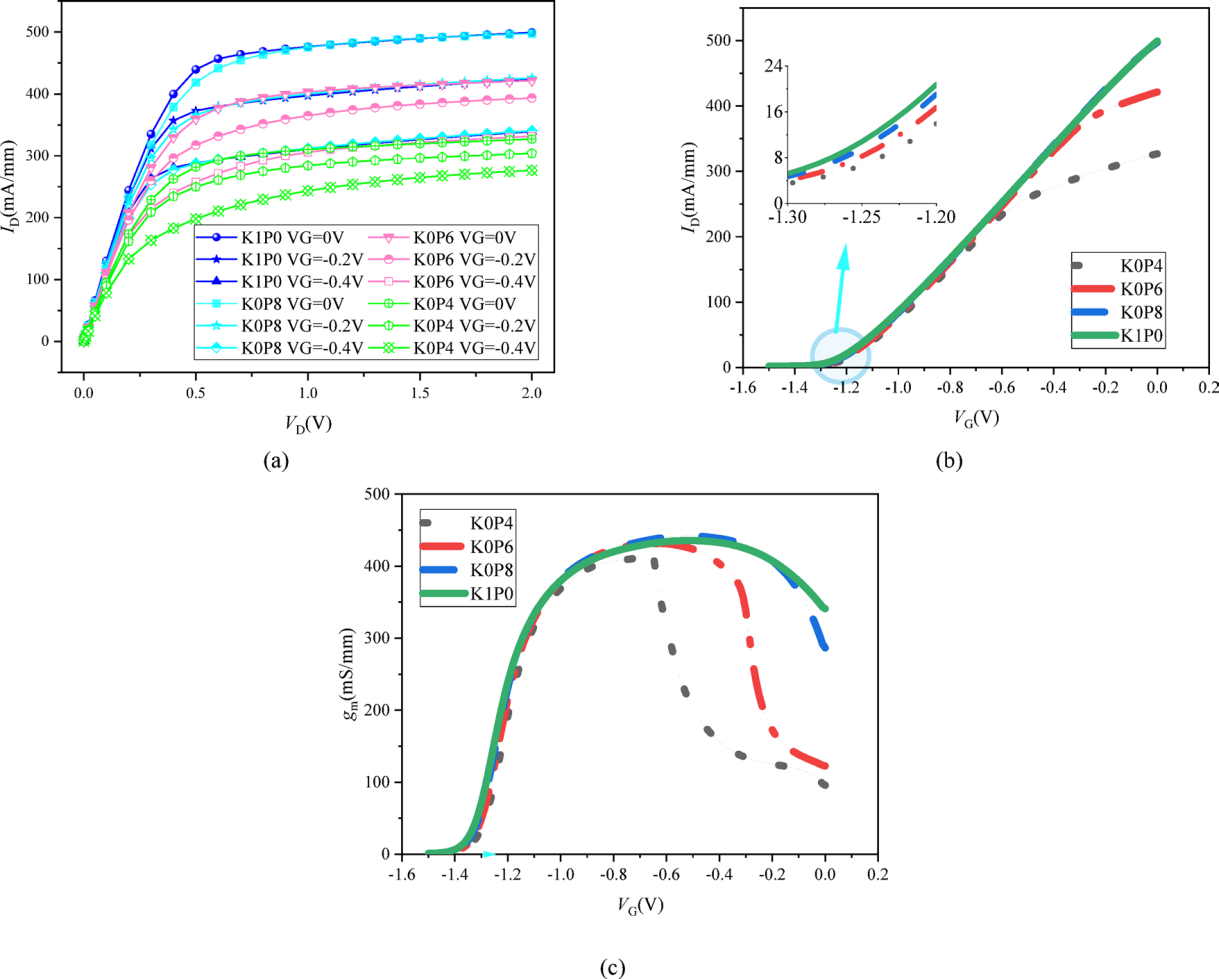
DC electrical characteristics highlighting the robustness–performance trade-off. **a** Output *I*_*D*_*–V*_D_ curves. **b** Transfer *I*_D_–*V*_G_ curves. **c** Transconductance *g*_m_–*V*_G_. Stable threshold voltage but degraded on-current and peak *g*_m_ are observed as doping shifts from the drain to the source side.

A systematic evaluation of the impact of lateral δ‑doping redistribution is performed by analyzing the DC output, transfer, and transconductance characteristics, comprehensively summarized in Fig. 6a–c.

Figure 6a presents the output characteristics (*I*_D_–*V*_D_) for the four schemes at *V*_G_ = 0, − 0.2, and − 0.4 V. A clear and consistent trend is observed: as the drain-side δ-doping is progressively weakened (K1P0 → K0P4), the drain current density decreases systematically across the entire *V*_D_ range for all gate biases. This reduction is evident from the outset in the linear region. For instance, at *V*_G_ = 0 V, the current at *V*_D_= 0.1 V is highest for K1P0 and lowest for K0P4. This behavior directly indicates that reducing the donor supply in the drain-side access segment increases the effective series resistance, thereby limiting the channel current delivery under identical gate-drive conditions.

The transfer characteristics (*I*_D_–*V*_G_) in Fig. 6b and the transconductance (*g*_m_–*V*_G_) in Fig. 6c further elucidate the electrostatic and transport effects. The transfer curves in Fig. 6b show that the threshold voltage (*V*_T_), as visible in the inset focusing on the turn-on region, exhibits only minor variation (within ~ 30 mV) across the four doping schemes. This result validates the core design principle: keeping the gate-under segment (Seg2) fixed successfully preserves the intrinsic gate-controlled electrostatics and the turn-on behavior.

In contrast, a clear performance trade-off is observed in the extrinsic conduction and gain metrics. The saturation current *I*_DSS_ (at *V*_G_ = 0 V) decreases monotonically from K1P0 to K0P4, as evident from the spacing of the curves in Fig. 6b. Concurrently, the peak transconductance (*g*_m, max_) in Fig. 6c shows a significant and consistent degradation with weaker drain-side doping, decreasing from the highest value for K1P0 to the lowest for K0P4. These trends collectively demonstrate that while the intrinsic channel control is maintained, the reduction of δ-doping in the drain-side access region introduces additional external resistance. This leads to a measurable degradation in the maximum current-drive capability, small-signal gain, and RF potential of the device^7^.

In summary, the DC analysis quantitatively reveals the direct consequence of the proposed doping redistribution strategy: it successfully maintains threshold stability at the cost of degraded conduction and gain. The following section will quantify this trade-off further (e.g., in terms of *R*_on·W_, and cutoff frequency, *f*_T_) and correlate it with the electric-field and robustness analysis.

### On‑resistance, small‑signal capacitances, and RF‑relevant metrics

**Fig. 7:**
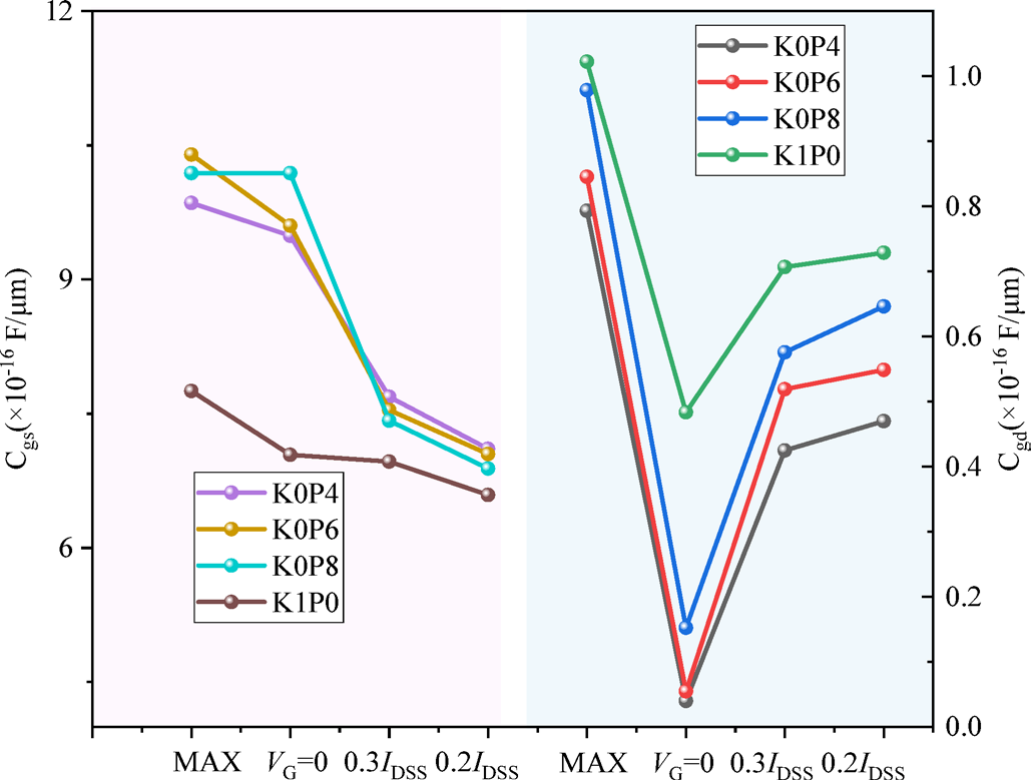
Comparison of small-signal capacitances at key operating points. Gate-source capacitance *C*_gs_ (left axis, ×10^−16^ F/µm) and gate-drain capacitance *C*_gd_ (right axis, ×10^−16^ F/µm) for the four redistribution schemes, extracted at the peak transconductance point (MAX), open-channel condition (*V*_G_ = 0 V), and at the 0.3*I*_DSS_and 0.2*I*_DSS_ operating points. The monotonic decrease in *C*_gd_ with reduced drain-side doping is evident, particularly at the 0.3*I*_DSS_ point.

**Fig. 8:**
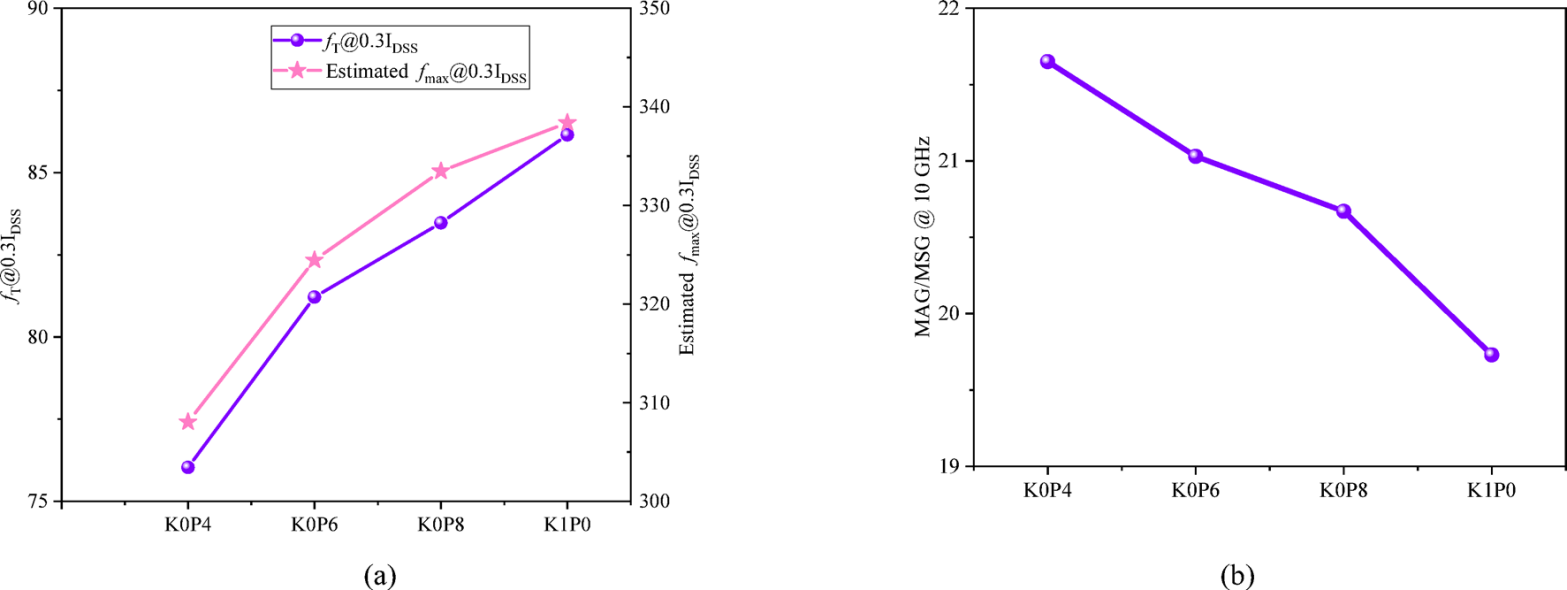
RF-relevant metrics for the four redistribution schemes. **a ***f*_T_@0.3*I*_DSS_ and estimated *f*_max_@0.3*I*_DSS_. Both frequency-limit indicators decrease as the lateral $\:\delta\:$-doping is progressively shifted from the drain side to the source side (K1P0 $\:\to\:$ K0P4). **b** MAG/MSG evaluated at 10 GHz under the same operating point.

The trade-off between robustness enhancement and RF/DC performance, induced by the lateral δ-doping redistribution, is quantitatively detailed in Figs. 7 and 8, and the unified metrics summarized in Table 4. All key parameters are evaluated at the consistent 0.3*I*_DSS_operating point to enable a fair and focused comparison.

The most direct electrical penalty is observed in the series resistance. The width-normalized on-resistance, *R*_on·W_, increases monotonically and substantially from 0.874 Ω·mm for the baseline K1P0 to 1.271 Ω·mm for K0P4, marking a 45.4% increase. This trend directly confirms that weakening the drain-side δ-doping depletes the access region, thereby increasing its parasitic resistance and limiting current delivery^8^.

The evolution of small-signal capacitances across key operating points, critical for understanding RF response and field modulation, is systematically presented in Fig. 7. A pronounced and consistent trend is observed for the gate-drain capacitance *C*_gd_. At the RF-relevant 0.3*I*_DSS_ point, *C*_gd_ shows a clear reduction from 0.71 × 10^−16^ F/µm for K1P0 to 0.43 × 10^−16^ F/µm for K0P4. This systematic decrease in *C*_gd_ is a direct signature of enhanced depletion and reduced charge coupling in the drain-side access region, which is intrinsically linked to the intended alleviation of gate-drain field crowding. The gate-source capacitance *C*_gs_ at 0.3*I*_DSS_ exhibits a more moderate increase.

The extrinsic small-signal gain at the 0.3*I*_DSS_ point, captured by the transconductance *g*_m_, shows a slight degradation from 415 mS/mm for K1P0 to 388 mS/mm for K0P4. This reduction, coupled with the increasing *R*_on·W_ and decreasing *C*_gd_, directly impacts the RF figure of merit.

Consequently, the current-gain cutoff frequency at 0.3*I*_DSS_ (*f*_T_@0.3*I*_DSS_) exhibits a clear downward trend, as distinctly plotted in Fig. 8a. It decreases from 86.1 GHz for K1P0 to 76.0 GHz for K0P4, corresponding to an 11.7% degradation. This indicates the RF performance penalty of the design strategy aimed at robustness improvement.

A similar trend is observed for the estimated *f*_max_@0.3*I*_DSS_, which decreases monotonically from 338.38 GHz for K1P0 to 333.48 GHz for K0P8, 324.46 GHz for K0P6, and 308.05 GHz for K0P4, corresponding to an overall reduction of approximately 8.9%. Although the absolute values of the estimated *f*_max_ are substantially higher than the corresponding *f*_T_ values, this does not by itself indicate a simulation or extraction error. In the present framework, the estimated *f*_max_ is extracted from the 0 dB crossing of the unilateral power gain and is used primarily as a comparative RF indicator. Accordingly, the main significance here lies in the consistent monotonic trend under identical extraction conditions, rather than in the absolute value itself.

By contrast, MAG/MSG evaluated at 10 GHz shows a moderate increase from 19.73 dB for K1P0 to 21.65 dB for K0P4, as shown in Fig. 8b. This indicates that the RF impact of lateral $\:\delta\:$-doping redistribution is metric dependent: while the frequency-limit indicators (*f*_T_ and estimated *f*_max_) degrade as robustness increases, the gain metric evaluated at a fixed moderate frequency does not follow the same penalty trend.

The overall electrical trade-off is summarized in Table 4. As the design moves from K1P0 to K0P4, *R*_on·W_ increases, C_gd_ decreases, *g*_m_ slightly degrades, and *f*_T_ drops, while the off-state robustness voltage *V*_crit_ is substantially improved. The estimated *f*_max_ shows a corresponding monotonic decrease, whereas MAG/MSG at 10 GHz exhibits a different trend. Overall, these results indicate that robustness-oriented charge redistribution improves the off-state high-voltage margin at the expense of measurable penalties in conduction loss and in the high-frequency-limit RF metrics.

**Table 4:** Summary of key resistive and RF-relevant metrics for the four lateral δ-doping redistribution schemes.

Scheme	*R*_on·W_ (Ω·mm)	C_gs_@ 0.3I_DSS_ (×10^−16^ F/µm)	C_gd_@ 0.3I_DSS_ (×10^−16^ F/µm)	g_m_@ 0.3I_DSS_ (mS/mm)	f_T_@0.3I_DSS_ (GHz)	Estimated f_max_@0.3I_DSS_ (GHz)	MAG/MSG @10 GHz (dB)	V_crit_(V)
K1P0	0.874	7.0	0.71	415	86.1	338.38	19.73	11.99
K0P8	0.921	7.4	0.58	419	83.5	333.48	20.67	13.28
K0P6	1.039	7.5	0.52	411	81.2	324.46	21.03	15.41
K0P4	1.271	7.7	0.43	388	76.0	308.05	21.65	19.06

*V*_crit_ is defined as the drain voltage at which |I_*D*_| = 1 × 10^−5^ A under *V*_G_ = -3 V.

### Electric‑field redistribution at the gate–drain edge (mechanistic evidence)

**Fig. 9:**
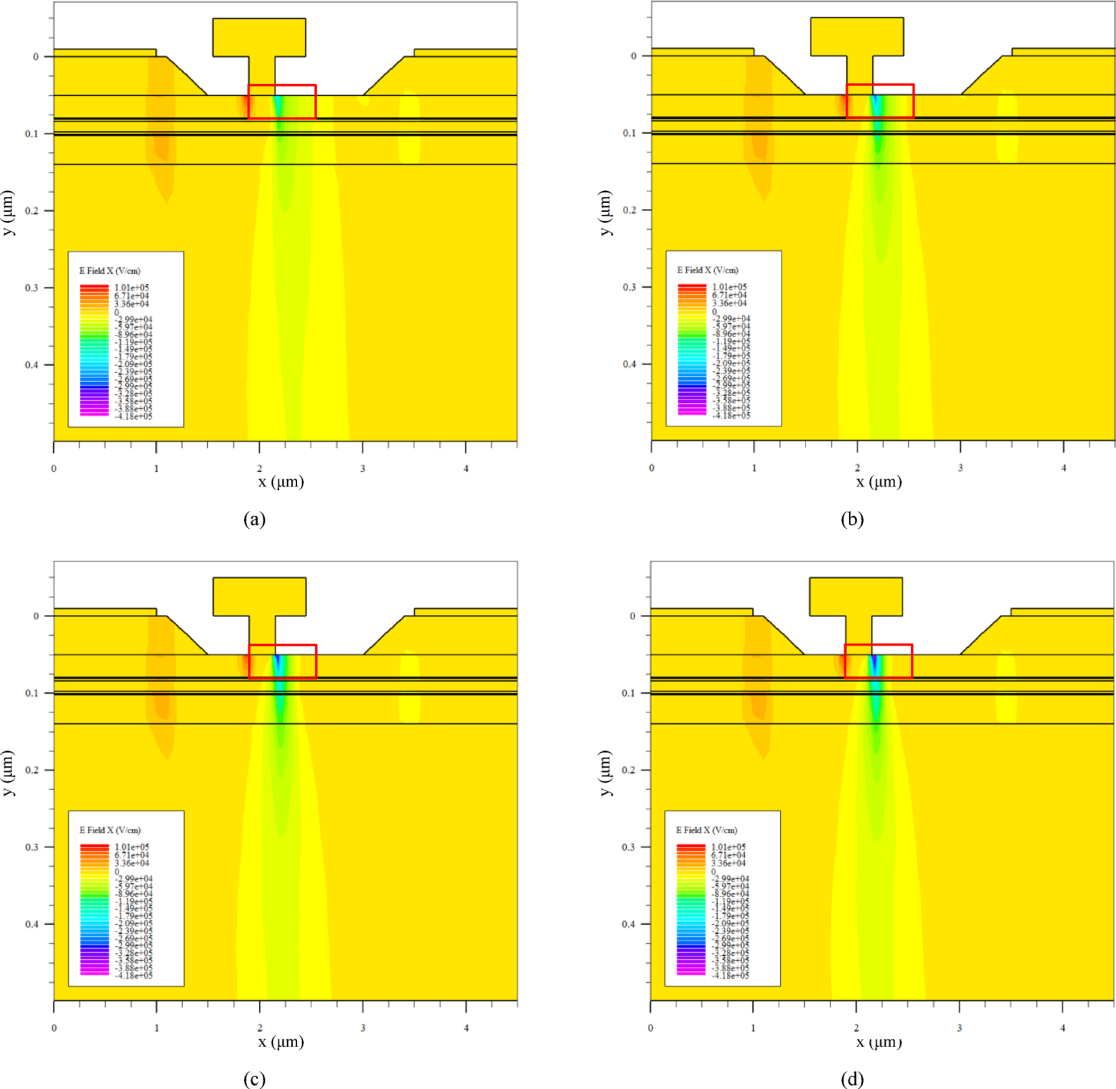
Two-dimensional contour maps of the lateral electric-field component *E*_x_ (V/cm) under *V*_G_ = − 0.6 V, *V*_D_= 3.0 V. **a** K1P0, **b** K0P8, **c** K0P6, **d** K0P4. A unified color scale highlights the progressive suppression and spatial spreading of the high-field region near the gate–drain edge as the drain-side δ-doping is reduced.

To provide direct visual and quantitative evidence for the intended field-relief effect, the lateral electric-field distribution *(E*_x_) under a common bias of *V*_G_ = − 0.6 V and *V*_D_= 3.0 V is analyzed for all four schemes. This bias condition represents a relevant semi-off-state operating point where field crowding is pronounced. Figure 9 presents the two-dimensional contour maps of *E*_x_ near the gate–drain edge, using a unified color scale across the four sub-figures [(a) K1P0, (b) K0P8, (c) K0P6, (d) K0P4] for direct comparison. A clear and systematic evolution is observed: the intensely concentrated high-field region directly beneath the gate edge becomes progressively suppressed and spatially more distributed towards the drain contact as the drain-side δ-doping (Seg3) is reduced from K1P0 to K0P4.

**Fig. 10:**
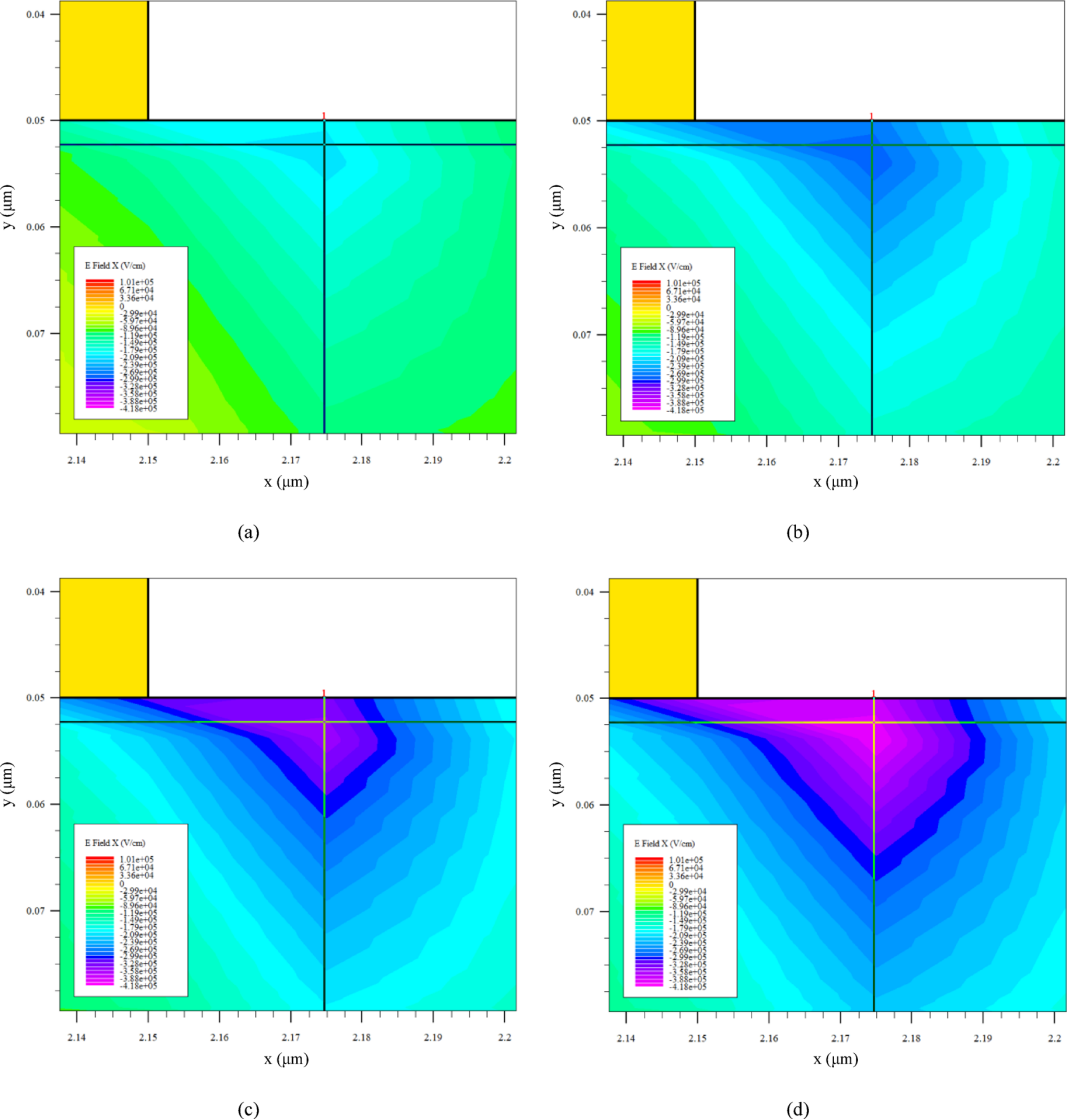

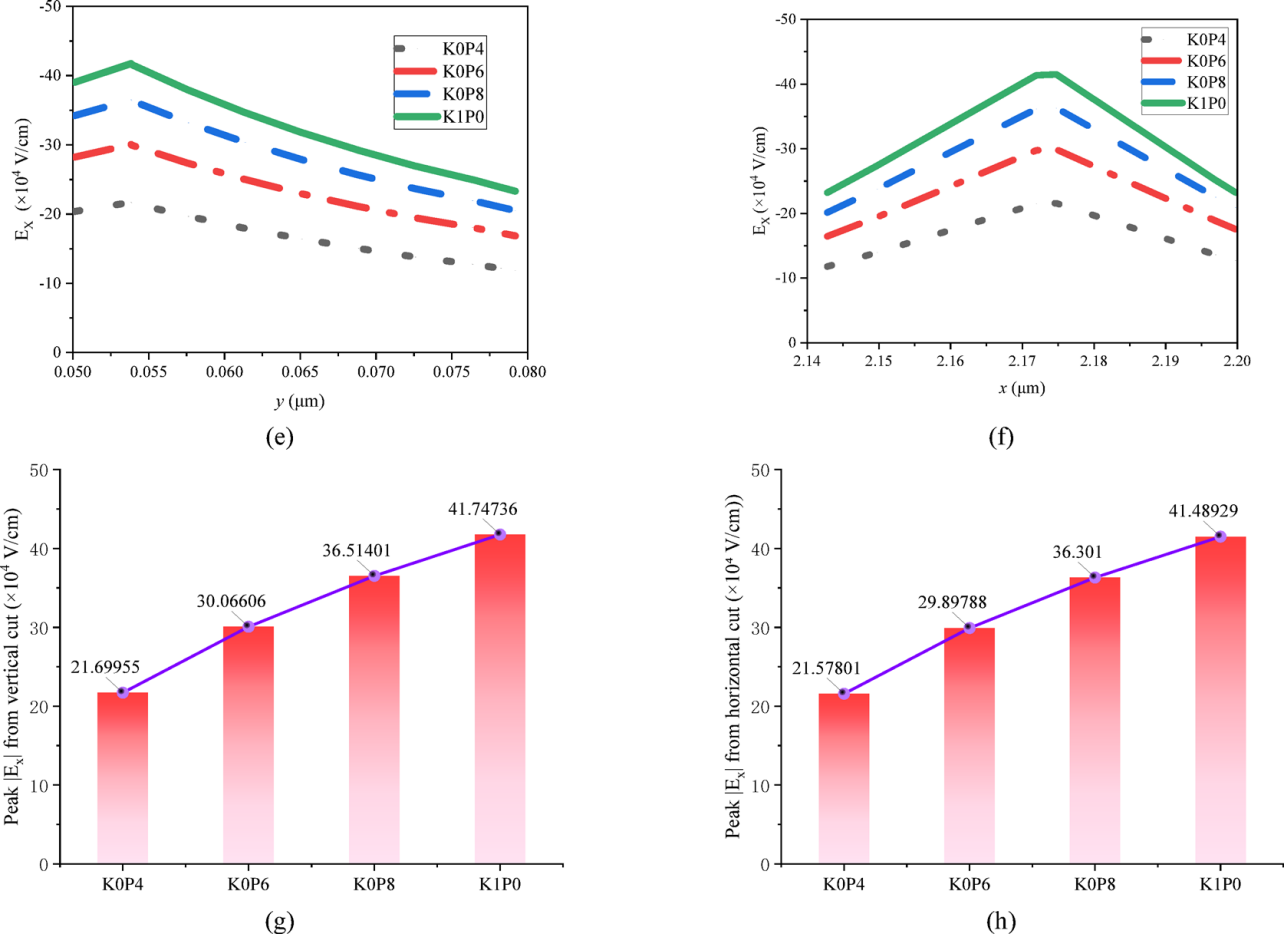
Detailed field analysis and line-cut profiles under *V*_G_ = − 0.6 V, *V*_D_= 3.0 V. **a**–**d** Magnified views of the gate–drain edge region corresponding to the four schemes in Fig. 9. **e** Vertical line-cut profiles of *E*_x_ at a fixed lateral position (*x* = 2.175 μm), showing the reduction in peak electric field magnitude. **f** Horizontal line-cut profiles of *E*_x_ at a fixed depth in the channel (*y* = 0.052 μm), illustrating the broadening and reduction of the lateral field distribution. **g** Extracted peak $\:\mid\:{E}_{x}\mid\:$values from the vertical line-cut analysis. **h** Extracted peak $\:\mid\:{E}_{x}\mid\:$ values from the horizontal line-cut analysis. Both plots show a monotonic decrease in the electric-field peak as the drain-side $\:\delta\:$-doping is reduced.

For detailed quantitative analysis, Fig. 10 provides magnified views and one-dimensional line-cut profiles. Sub-figure 10a–d show enlarged depictions of the high-field region from Fig. 9, further highlighting the reduction in peak field intensity. The mechanistic evidence is further supported by the line cuts: Fig. 10e plots *E*_x_ along a vertical cut at *x* = 2.175 μm (traversing the gate–drain edge), demonstrating a systematic lowering of the peak field magnitude with reduced Seg3 doping. Correspondingly, Fig. 10f shows *E*_x_ along a horizontal cut in the channel at *y* = 0.052 μm, revealing that the lateral extent of the high-field region is reduced and the field profile becomes less steep for schemes with weaker drain-side doping. To make the relation between $\:\delta\:$-doping redistribution and the electric-field peak more explicit, Fig. 10g and h summarize the extracted peak $\:\mid\:{E}_{x}\mid\:$ values from the vertical and horizontal line-cut analyses, respectively. In both cases, a clear monotonic decrease in the peak $\:\mid\:{E}_{x}\mid\:$ is observed as the drain-side $\:\delta\:$-doping is progressively reduced from K1P0 to K0P4, indicating a negative correlation between the drain-side $\:\delta\:$-doping level and the electric-field peak near the gate–drain edge.

This observed field redistribution provides the direct physical link to the electrical trends in Sect. “On‑resistance, small‑signal capacitances, and RF‑relevant metrics”. The suppressed and broadened *E*_x_ profile is a direct consequence of enhanced depletion in the drain access region due to reduced δ-doping. This depletion effect simultaneously explains the reduction in *C*_gd_ (reduced gate-drain capacitive coupling) and the increase in *R*_on_ (increased access resistance). Therefore, the electric-field analysis in Figs. 9 and 10 provides mechanistic support for the interpretation that lateral $\:\delta\:$-doping redistribution mitigates gate–drain field crowding. More specifically, reducing the donor supply in the drain-side access region enhances local depletion and redistributes the longitudinal potential drop, thereby relieving the electric-field peak at the gate–drain edge. Within the present framework, this interpretation is consistent with the observed improvement in *V*_crit_.

### Off-state robustness quantified by a reproducible metric *V*_crit_

The primary objective of the lateral δ-doping redistribution is to enhance the device’s high-voltage robustness. As discussed in Sect. “Off-state robustness definition and extraction of *V*_crit_”, directly predicting absolute avalanche breakdown voltage from TCAD can be sensitive to model specifics. Therefore, we employ a practical and reproducible off-state robustness metric, *V*_*crit*_, defined as the applied drain voltage *(V*_D_) at which the magnitude of the drain current reaches |*I*_D_| = 1 × 10^−5^ A under a fixed, deep gate-off bias of *V*_G_ = − 3 V. This criterion provides a stable basis for comparing the relative high-voltage margins across different design schemes.

**Fig. 11:**
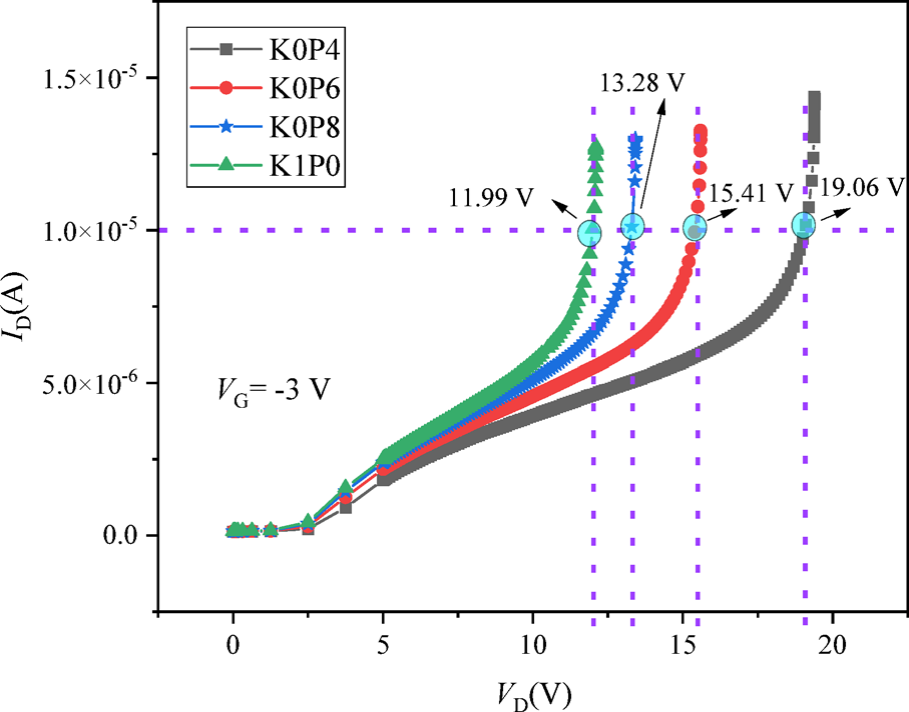
Off-state *I*_*D*_*–V*_D_ characteristics at *V*_G_ = − 3 V and extraction of *V*_crit_. The drain current magnitude |*I*_D_| is plotted against the applied drain voltage *V*_D_. The horizontal dashed line indicates the fixed current threshold of 1 × 10^−5^ A. The *V*_crit_ for each scheme is defined by the *V*_D_ value at which its curve intersects this threshold.

The extraction of *V*_*crit*_ *is* illustrated in Fig. 11, which plots the off-state *I*_*D*_*–V*_D_ characteristics for all four schemes. A clear, monotonic rightward shift of the curves to higher drain voltages is observed as the drain-side doping is reduced. The corresponding *V*_crit_ values, extracted at the intersection of each curve with the 10^−5^ A threshold (dashed line), are:

- 11.99 V for the baseline K1P0,
- 13.28 V for K0P8,
- 15.41 V for K0P6,
- 19.06 V for K0P4.

This represents a substantial and monotonic enhancement in robustness, with *V*_crit_ increasing by approximately 10.8%, 28.5%, and 58.9% for K0P8, K0P6, and K0P4, respectively, relative to the K1P0 baseline. This direct electrical evidence is fully consistent with the field-mitigation observed in the section “Electric‑field redistribution at the gate–drain edge (mechanistic evidence)”, confirming that the reduction of δ-doping in the drain-side access region (Seg3) effectively alleviates electric-field crowding, thereby requiring a higher applied drain bias to generate the same off-state leakage current.

### Robustness–performance trade‑off analysis

The comprehensive dataset from the sections  “DC characteristics: output, transfer, and transconductance behavior”, “On‑resistance, small‑signal capacitances, and RF‑relevant metrics”, “Electric‑field redistribution at the gate–drain edge (mechanistic evidence)” and “Off-state robustness definition and extraction of *V*_crit_” quantifies a clear trade-off engineered by the lateral δ-doping redistribution: enhanced off-state robustness is achieved at the expense of degraded DC and RF performance.

**Fig. 12:**
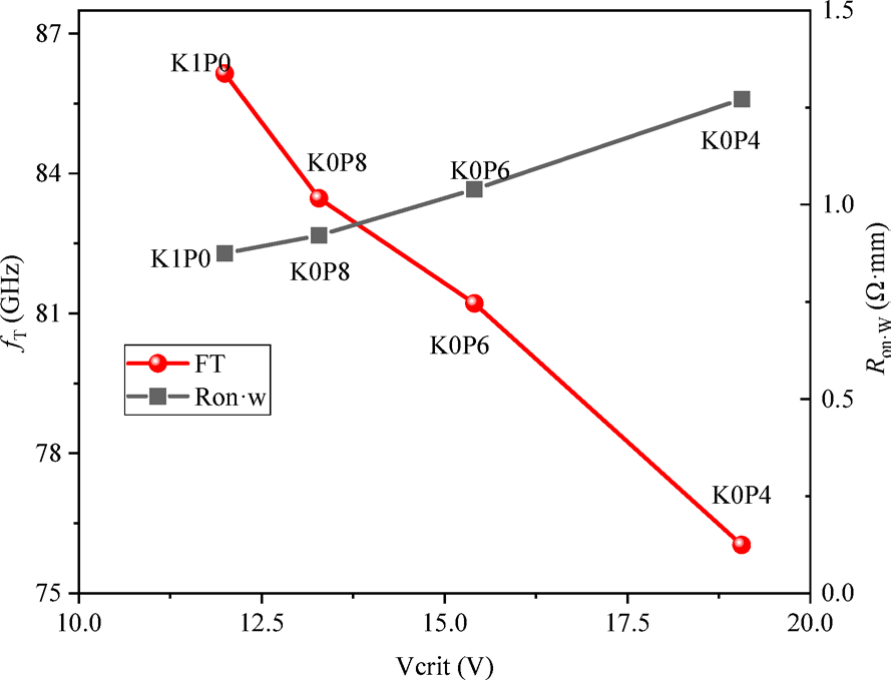
Robustness–performance trade-off map. The off-state robustness voltage (*V*_crit_) is plotted against the RF cutoff frequency (*f*_T_@0.3*I*_DSS_, left axis, red circles) and the normalized on-resistance (*R*_on·W_, right axis, black squares) for the four redistribution schemes. Arrows indicate the direction of increasing drain-side δ-doping reduction (K1P0 → K0P4).

This fundamental trade-off is visualized in the design map shown in Fig. 12, where the robustness metric *V*_*crit*_ is plotted against the two primary performance penalties: the RF cutoff frequency *(f*_T_@0.3*I*_DSS_) and the width-normalized on-resistance (*R*_on·W_). The plot crystallizes the design landscape: moving from the K1P0 baseline towards K0P4 (i.e., progressively reducing drain-side doping) results in a significant, monotonic increase in *V*_crit_ (from 12.0 V to 19.1 V). Concurrently, this robustness gain is accompanied by a monotonic degradation in *f*_*T*_*(*from 86.1 GHz down to 76.0 GHz) and an increase in *R*_on·W_ (from 0.87 Ω·mm up to 1.27 Ω·mm). Within the explored design space, higher robustness is therefore accompanied by greater penalties in speed and conduction loss.

From a comparative perspective, the four schemes define a monotonic trade-off trend within the explored design space. K0P4 shows the largest improvement in the comparative robustness metric *V*_crit_, together with the largest penalties in *f*_T_ and *R*_on·W_, whereas K0P8 shows a smaller robustness gain with correspondingly smaller DC/RF penalties. K1P0 serves as the segmented-uniform baseline for comparison.

Overall, the three-segment lateral δ-doping redistribution strategy serves as a useful comparative design variable for analyzing the robustness–performance trade-off in GaAs pHEMTs within the present simulation framework. The results indicate that weaker drain-side δ-doping tends to improve the comparative robustness metric *V*_crit_ while incurring penalties in conduction loss and high-frequency performance. Further work, including experimental validation and co-optimization with geometric or field-management approaches, is needed before translating these comparative trends into broader device-level design guidance.

## Conclusions

### Summary of findings

This work investigated a three-segment lateral $\:\delta\:$-doping redistribution strategy for GaAs pHEMTs, in which the gate-under segment was kept unchanged, while the source-side and drain-side segments were systematically redistributed under an approximately conserved total length-weighted lateral δ-dose constraint. Under otherwise identical device geometry, material composition, and physical-model settings, a controlled comparison was performed to evaluate how lateral $\:\delta\:$-doping redistribution affects electric-field distribution, DC/RF characteristics, and off-state robustness.

The results show that reducing the drain-side $\:\delta\:$-doping progressively relieves electric-field crowding near the gate–drain edge. Both the two-dimensional field maps and the extracted line-cut results indicate a monotonic reduction in the peak $\:\mid\:{E}_{x}\mid\:$as the drain-side $\:\delta\:$-doping is weakened, **s**howing a clear monotonic relation between lateral $\:\delta\:$-doping redistribution and electric-field peak suppression. This field-relief effect is consistent with the observed improvement in the comparative robustness metric *V*_crit_, which increases monotonically from 11.99 V for K1P0 to 13.28 V for K0P8, 15.41 V for K0P6, and 19.06 V for K0P4.

The robustness improvement is accompanied by measurable electrical penalties. As the design shifts from K1P0 to K0P4, the width-normalized on-resistance *R*_on__·__W_ increases from 0.874 to 1.271 $\:{\Omega\:}\cdot\:$mm, while the current-gain cutoff frequency *f*_T_@0.3*I*_DSS_ decreases from 86.1 to 76.0 GHz. The estimated *f*_max_@0.3*I*_DSS_, used here as a comparative RF indicator, shows a corresponding monotonic decrease from 338.38 to 308.05 GHz, indicating that the high-frequency-limit RF metrics are degraded as robustness increases. By contrast, MAG/MSG evaluated at 10 GHz exhibits a different trend, showing that the RF impact of lateral $\:\delta\:$-doping redistribution is metric dependent. Overall, these results indicate that the proposed redistribution strategy improves the off-state high-voltage margin at the expense of increased conduction loss and reduced high-frequency RF performance.

Within the explored design space, the present study provides a quantitative comparative basis for evaluating the robustness-performance trade-off associated with lateral $\:\delta\:$-doping redistribution in GaAs pHEMTs.

### Limitations and future work

The present study is based on a two-dimensional TCAD framework and is intended to provide a controlled comparative evaluation of lateral $\:\delta\:$-doping redistribution in GaAs pHEMTs. Accordingly, the reported results should be interpreted primarily in terms of relative trends within the explored design space. In particular, *V*_crit_ is used here as a practical comparative robustness metric under a fixed off-state criterion, rather than as a calibrated absolute breakdown voltage. Experimental validation and further calibration against measured DC/RF and high-voltage characteristics will therefore be important in future work. In addition, future studies may extend the present framework by combining lateral $\:\delta\:$-doping redistribution with geometry optimization and other field-management approaches.

## Data Availability

The datasets used and/or analysed during the current study available from the corresponding author on reasonable request.
